# A novel treatment approach for a patient with advanced low rectal cancer: A case report and literature review

**DOI:** 10.1097/MD.0000000000033497

**Published:** 2022-04-07

**Authors:** Xuejun Jiang, Feng Zhou, Fangjun Yuan, Xinyi Lei, Cui Liu, Zujin Ji

**Affiliations:** a Department of Colorectal and Anal Surgery, Sinopharm Dongfeng General Hospital, Hubei University of Medicine, Shiyan, Hubei, PR China.

**Keywords:** conservative treatment, cryosurgery, organ-sparing treatments, rectal neoplasm, surgical procedures

## Abstract

In the treatment of low rectal cancer (LRC), preserving the anal sphincter is increasingly attracting the attention of colorectal surgeons. Many patients refused to perform a colostomy. Here, we report a case of LRC in a middle-aged woman and the clinical implications of the symptom, the treatment process of LRC, and the complications.

**Patient concerns:**

A 46-year-old woman visited our department with a tumor found on her physical examination because of hemafecia. Then she refused to perform abdominoperineal resection.

**Diagnosis:**

The patient first completed a colonoscopy and then underwent a rectal biopsy. The tumor was diagnosed as a rectal adenocarcinoma after pathological evaluation. Then it was staged by magnetic resonance imaging and enhanced computed X-ray tomography.

**Interventions:**

The treatment consisted of chemoradiotherapy followed by cryoablation.

**Outcomes:**

The patient achieved a good oncological outcome and preserved the sphincter successfully. The post-cryoablation course of the patient was uneventful and he remained healthy at the 1-year follow-up.

**Lessons:**

The preservation of anal sphincters has attracted more and more attention from colorectal surgeons. From the patient's perspective, the preservation of the anal sphincter was a key part of her treatment. We should try to meet the wishes of patients on the basis of curing the disease.

## 1. Introduction

Despite neoadjuvant chemoradiotherapy (CRT) improves sphincter preservation rate for patients with low rectal cancer (LRC). Many patients still necessitate performing abdominoperineal resection (APR) which results in a permanent stoma and impaired Quality of Life (QoL).^[1]^ Parts of these patients refused to operate with APR for eschewing stomas, as a result, disease progress may threaten their lives. Sphincter and QoL preservation have been the focuses of LRC treatment. Cryotherapy as a local ablation approach has been proposed to treat tumors, including liver cancer, prostate cancer, and skin lesions.^[2]^ Cryotherapy has also been used as a treatment after malignant rectal bleeding.^[3]^ To preserve the sphincter and QoL, a novel treatment approach was introduced to a patient with advanced LRC. This paper reported a case treated with neoadjuvant CRT combined with cryotherapy to meet her sphincter preservation expectations.

## 2. Demographic information and clinical manifestation

A 46-year woman was diagnosed with LRC. Clinical manifestations were common, including hematochezia and sensation of rectal tenesmus. The result of the physical examination was described as that a hard protuberant tumor can be palpated on the back wall of the rectum at a distance of 3 to 6 cm from the anal edge, accounting for about one-third of the intestinal circumference, with tumor fixation.

## 3. Diagnostic assessment

**Figure 1.**
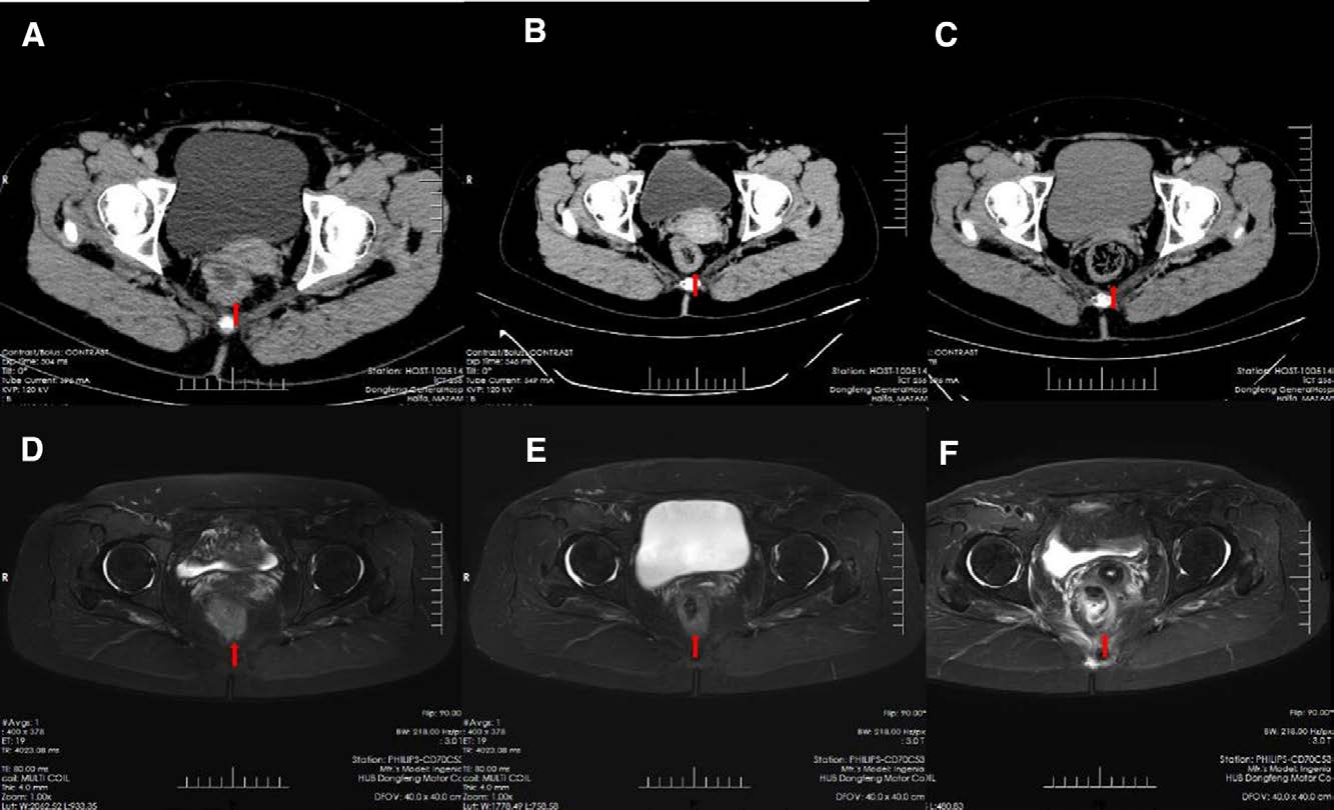
(A) November 2021, enhanced CT showed that the wall of the lower segment of the rectum was unevenly thickened, the mucosa surface was slightly enhanced, and the serous surface was still smooth before treatment. (B) March 2022, after chemoradiotherapy, CT showed that the middle and lower rectal wall was thickened, the mucosa surface was enhanced, and the serous surface was still smooth before cryotherapy. (C) May 2022, after cryotherapy, the middle and lower rectal wall of CT was slightly thickened, the mucosa surface was slightly enhanced, and no obvious tumor was present. (D) November 2021, enhanced MRI sequences of the pelvic cavity showed that the intestinal wall of the middle and lower segments of the rectum was unevenly thickened at a distance of 3.2 cm from the anus, the T2 lipo-pressing sequence showed equal and high signal shadows, the length of which was about 5.0 cm, the serous surface was slightly rough before treatment. (E) March 2022, after chemoradiotherapy, pelvic MR enhanced each sequence showed that the middle and lower segments of the rectum were slightly thickened, the T2 fat pressure sequence showed equal and high signal shadow, the surface of the intestinal wall was smooth, and the enhanced scan of intravenous Magnevist showed slight enhancement before cryotherapy. (F) May 2022, the sequence of enhanced pelvic MR scanning showed that the lower rectal wall was slightly thickened after chemotherapy for rectal cancer, the T2 lipo-pressure sequence showed equal signal shadow, and the surface of the intestinal wall was smooth. CT = computed tomography, MRI = magnetic resonance imaging.

**Figure 2.**
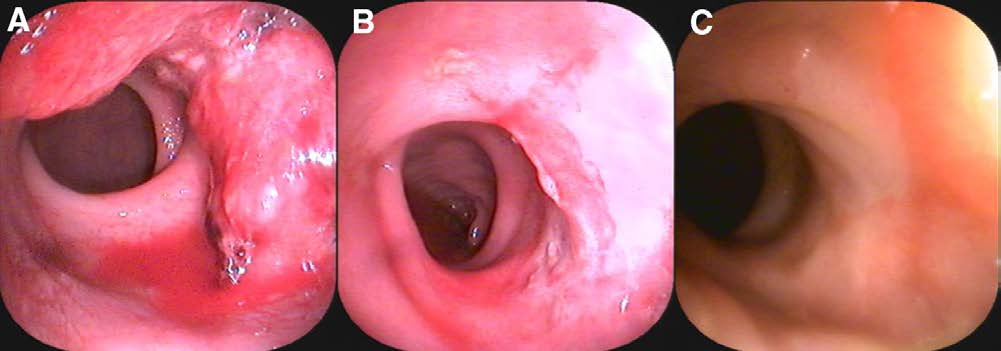
(A) The colonoscopy image displayed the ulcerative tumor occupied 1/3 of the intestinal cavity before treatment. (B) After treatment, the colonoscopy image showed that the tumor disappeared 2 months later, and inflammatory granulation tissue was visible in the rectal wall. (C) The colonoscopy image indicated the scar was visible on the rectal wall, and no ulcerative lesion or tumor was seen after cryoablation half year later.

Laboratory investigation results, including tumor markers, were unremarkable. Tumor node metastasis classification system was adopted to stage the tumor, the challenge of diagnosis was that we cannot stage accurately by imaging examination. Magnetic resonance imaging (MRI) and enhanced computed tomography (CT) were employed in this patient, and she was staged into cT3N2aM0(Fig. 1). Electronic colonoscopy showed that the tumor occupied one-third of the intestinal lumen (Fig. 2).

## 4. Therapeutic interventions

**Figure 3.**
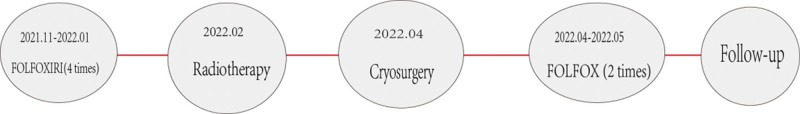
The timeline of the treatment process. FOLFOXIRI = (Oxaliplatin Day 1, Irinotecan Day 1, Fluorouracil Day 1–2, Calcium folinate Day 1–2); Radiotherapy = (GTVnd 5000Cgy/25F, CTV 4500Cgy/25F); FOLFOX = (Oxaliplatin Day 1, fluorouracil Day 1–2, calcium folinate Day 1–2). CTV = clinical target volume; GTVnd = gross tumor volume of metastatic lymph nodes.

To achieve a good oncological outcome, we chose to conduct neoadjuvant CRT, the specific scheme was selected according to the guideline (Fig. 3). After neoadjuvant CRT, part of the solid tumor can still be touched by rectal palpation, APR needed to be performed with this case, but she refused to operate with this procedure because of a stoma. We chose to perform cryosurgery to preserve sphincters after a discussion with Multidisciplinary Diagnosis Team. Intestinal cleanser was not distributed for oral use for treatment preparation, and only an enema was performed the day before cryotherapy. The key technical parameters were cryo-time (720 s), cryo-depth (beyond the edge of tumor 5 mm), freeze-thaw cycle (2 cycles), and cryo-diameter(4 cm) during the period of cryoablation. The important milestones related to diagnoses and interventions (Fig. 3).

## 5. Follow-up and outcomes

After 1 year follow-up, we could palpate a scar on the right posterior wall of 4 cm from the anal verge by digital rectal examination. The imaging examination indicated that there was no tumor recurrence to date (Figs.1 and 2). Then we conducted pathological examination by biopsy in situ a month later after cryoablation, and the result indicated that inflammatory granulation tissue replaced the ulceration, no tumor lesion, and no dysplasia post-cryoablation.

The patient had good intervention adherence and tolerability during the period of CRT and cryosurgery. The physical status was assessed by Eastern Cooperative Oncology Group score (Grade 0), and the function status by KPS (90 marks). The result showed that there was no obvious difference in physical and functional status before and after therapy.

During the period of chemotherapy, the adverse drug reaction includes nausea, vomiting, and leukopenia (Grade II, according to WHO classification criteria for toxicity and side effects of chemotherapy). We detected the adverse and unanticipated events after the cryotherapy. The most important complication was ulcer which was presented as expected. Considering the influence of CRT, the healing time persisted for 4 months, consequently, the patient had anal pain for nearly 4 months. It could be classed into 2 grades (Clavin–Dingo system) and cured by analgesics.

## 6. Discussion

Surgical procedure is the main approach for local rectal cancer.^[4]^ For local advanced LRC, standard treatment consisting of CRT followed by radical surgery results in poor functional results or stoma.^[5]^ Because of this reason, sphincter preservation for patients with ultra-LRC is a hot subject of cancer therapy. After the treatment of CRT, the Watch and Wait strategy seems to be an option, but the other patients still need local resection or total mesorectal excision. For early stages, radical resection could be avoided in favor of conservative approaches combining radiotherapy with or without chemotherapy, for good responders, to a local excision with a view of organ-preservation strategies.^[6]^ Despite surgeons having many tricks to preserve the sphincter, it is still impossible for all patients to avoid APR after CRT. Cryosurgery seems to be a reliable local treatment option to cure these cases. It was widely used in the treatment of tumors, such as breast cancer, prostate cancer, skin lesions, and liver cancer.^[7–10]^ The mechanism of this procedure is that the ultra-low temperature can cause cell necrosis and apoptosis.^[2]^ In order to achieve the goal of sphincter preservation, cryosurgery was employed in this patient post-CRT. It offers a new treatment option for patients with LRC, especially for those with a strong intention to preserve sphincters.

First, she had a strong aspiration to preserve the sphincter and refuse to perform a colostomy.

In this case, the patient stuck to preserve her anus. A patient decision aid was used to help our patient to make a decision. We took standard therapy which included concurrent CRT followed by cryosurgery and adjuvant chemotherapy for this patient.^[11]^ The tumor with additional downsizing allows sphincter-preserving surgery after the treatment.^[12]^ The choice of neoadjuvant CRT fellow the guidelines and was determined according to the opinions of the Multidisciplinary Diagnosis Team.^[13]^ The specific therapeutic schedule was illustrated in the timeline chart (Fig. 3). However, after preoperative neoadjuvant CRT, the patient still needed to perform APR by the evaluation of MRI and enhanced CT (Fig. 1). Given that sphincter preservation was a key point in our case, so we introduced cryoablation into this patient.

Second, we achieved a good oncological outcome.

After 1 year of follow-up, MRI and enhanced CT showed an equal signal shadow and smooth intestinal wall (Fig. 1). The result revealed that the tumor has no recurrence in situ which brought confidence to her. However, considering that cryoablation is a local treatment, it cannot ablate lymph node infiltration. Therefore, we conducted an intensive surveillance strategy for the patient to examine MRI and enhanced CT examination every 3 months as the follow-up strategy to ensure no recurrence.

Third, there was no severe complication.

Rectum is adjacent to the urethra or vagina. The ultra-low temperature resulted in ice balls on the intestinal wall, then tumor tissue formed an ulcer after the necrotic tissue abscised. The risk of ulcers included bleeding, the discomfort of being in tenesmus, and cicatricial constriction. In our case, there were no perioperative complications of grade 2 or above according to the Clavien–Dindo classification. In theory, given that cryoablation has a destructive effect on tumors in vivo, the complications may include rectovaginal fistula, rectourethral fistula, perianal infection, and cryo-shock. But it can be detected by real-time monitoring with ultrasound or CT to prevent fistula.

The preservation of the anal sphincter has attracted more and more attention from colorectal surgeons. In patients with risk factors such as depth of mesorectal invasion, extramural vascular invasion, or mesorectal fascia involvement, neoadjuvant CRT can reduce the risk of local tumor recurrence.^[14]^ MR images can be used to predict treatment response after CRT.^[15]^ For those with partial response, cryosurgery seems to be a promising treatment. It is more effective and secure for target lesions than other thermal ablation methods.^[16]^ In comparison with total mesorectal excision, the revisit strategy should be more frequent.

## 7. Patient perspective

From the patient's perspective, she is satisfied with the treatment of cryotherapy because of its good effect on oncological outcomes and sphincter preservation. This treatment saved her life and improved her QoL. On the one hand, she had no stoma to care for and avoided a lot of inconveniences. On the other hand, she could participate in various social activities and feel confident with her life. As she said, she would rather die of cancer than live with a stoma, this treatment met the expectations of preserving sphincter and improving the QoL.

## 8. Conclusions

The treatment consisting of CRT followed by cryoablation can be an option for patients with local advanced LRCs, it is mainly suitable for those with strong expectations to preserve sphincters.

## Author contributions

**Conceptualization:** Jiang Xuejun, Fangjun Yuan.

**Data curation:** Jiang Xuejun.

**Formal analysis:** Jiang Xuejun.

**Funding acquisition:** Jiang Xuejun, Fangjun Yuan.

**Investigation:** Feng Zhou.

**Methodology:** Feng Zhou, Cui Liu.

**Project administration:** Fangjun Yuan, Cui Liu.

**Resources:** Xinyi Lei, Cui Liu, Zujin Ji.

**Software:** Xinyi Lei, Cui Liu, Zujin Ji.

**Supervision:** Xinyi Lei, Cui Liu, Zujin Ji.

**Validation:** Xinyi Lei, Zujin Ji.

**Visualization:** Fangjun Yuan, Zujin Ji.

**Writing – original draft:** Fangjun Yuan, Xinyi Lei, Zujin Ji.

**Writing – review & editing:** Xinyi Lei, Zujin Ji.
